# Supplementary material for: Awareness and Practices towards Vaccinating Their Children against COVID-19: A Cross-Sectional Study among Pakistani Parents
Source: Healthcare (Basel). 2023 Aug 23;11(17):2378. doi: 10.3390/healthcare11172378 (PMC10487428; doi:10.3390/healthcare11172378)
Supplement: Supplementary file 1 [file healthcare-11-02378-s001.zip › healthcare-2515950-supplementary.pdf]

Awareness and Practices towards vaccinating their children against COVID-19: A Cross-Sectional study among Pakistani parents

Q1 Do you have any children?

1 Yes

2 No

Q2 Do you children age between 5-18 years

1 Yes

2 No

<u>Section 1: General information</u>

Q1 How many children do you have?

1 1

2 2

3 3

4 4 or more

Q2 Participant age

1 18-29

2 30-39

3 40-49

4 50-59

5 60 or more

Q3 Participant's sex?

1 Female

2 Male

Q4 Do you smoke?

1 Yes

2 No

3 Ex-smokers

Q5 Education level?

1 Illiterate

2 Religious education

3 Primary school

4 High school

5 Higher secondary education

6 Diploma

7 Bachelor’s degree

8 Postgraduation

Q6 Household average monthly income?

1 Less than 30000 PKR

2 31000-60000 PKR

3 More than 60000 PKR

Q7 Are you working /studying in a medical field?

1 Yes

2 No

Q8 Did you take COVID-19 vaccine?

1 Fully

2 Partially (one dose)

3 No

(If answered no go to the next question, if answered yes go to the next section (health status))

Q9 Are you willing to take the vaccine against COVID-19?

1 Yes

2 No

3 Maybe

Section 2: Health status of parents and children

Q1 Do you have a chronic disease? (Diabetes, hypertension, cardiac etc.).

1 Yes

No

Q2 Do you have any preterm children?

1 Yes

2 No

Q3 Do any of your children suffer from the following diseases (asthma, allergies, diabetes, cancer, cardiovascular disease, sickle cell anemia, liver disease and thalassemia) or taking steroids or immunosuppressant's medications?

1 Yes

2 No

Section 3: Experience with COVID-19

Q1 Do you know somebody close to you that was infected with COVID-19?

1 Yes

2 No

3 Maybe

Q2 Have you ever been infected with COVID-19?

1 Yes

2 No

3 Maybe

Q3 Has any of your children ever been infected with COVID-19?

1 Yes

2 No

3 Maybe

<u>Section 4: Your impressions towards COVID-19</u>

Q1 Estimate seriousness of COVID-19 on participants?

1 (low risk)

5 (high risk)

Q2 In your opinion, what is the likelihood that you will be infected with COVID-19 during the next 6 months?

1 I think that I will be infected and my symptoms will be severe

2 I don't know

3 I think that I will be infected and my symptoms will be mild

4 I do not think that I will be infected

Q3 Estimate seriousness of COVID-19 on child?

1 (Low risk)
2 (high risk)

Q4 In your opinion, what is the likelihood that your children will be infected with COVID-19 during the next 6 months?

1 I think that my child will be infected and his symptoms will be severe

2 I don't know

3 I think that my child will be infected and his symptoms will be mild

4 I do not think that my child will be infected

<u>Section 5: Knowledge towards COVID-19</u>

What are the symptoms of COVID19?

**symptoms**	**Yes**	**No**
Q1 Fever		
Q2 Chills		
Q3 Diarrhea		
Q4 Cough		
Q5 Otitis media^		
Q6 Loss of smell and taste senses		
Q7 No symptoms		

What procedures do you think may prevent COVID-19 infection?

**Procedures**	**Yes**	**No**
Q8 Washing hands with regular soap		
Q9 Using detergents		
Q10 Social distancing		
Q11 Avoid touching face/mouth/nose/eyes		
Q12 Avoid eating meat		
Q13 Consume herbs		

Q14 **Is there currently a drug in the pharmacies and hospitals that cure COVID-19 completely?**

1 Yes

2 No

3 Maybe

How COVID19 can be transmitted?

**Statement**	**Yes**	**No**
Q15 Drinking unclean water		
Q16 Eating unclean food		
Q17 Inhalation of respiratory droplets of infected person		
Q18 Eating or touching wild animals		

***What procedures have you taken to protect yourself and your children from COVID19?**

	All of the time	Most of the time	Sometimes	Rarely	Never
Q19 Wearing face masks					
Q20 Washing hands with regular soap					
Q21 Using detergents					
Q22 Social distancing					

Q23	Avoid touching face/mouth/nose/eyes					

Section 6: Knowledge towards COVID-19 Vaccines:

Q1 How effective is the use of COVID-19 vaccine for your children?

1 1(low efficacy)

2 5(high efficacy)

Q2 How safe is the use of COVID-19 vaccine for your children?

1 1(low efficacy)

2 5(high efficacy)

Q3 What are the common side effects of COVID-19 vaccine? (You can choose more than one answer)

1 Headache

2 High temperature

3 Chills

4 Pain at the site of injection

5 Swelling and redness at the site of injection (you can choose more than one answer)

6 General fatigue

7 I don't know

Q4 How is the vaccine administrated?

1 Intravenous injection

2 Intramuscular injection

3 Orally

4 I don't know

Section 7: willingness to vaccinate your children

Q1 Have all of your children between 5-18 years been vaccinated against COVID-19?

1 Yes

2 No

(If answered No go to the next question, if answered is yes automatically submit the questionnaire)

Q2 Are you willing to vaccinate your children against COVID-19?

1 Yes

2 No

(If the answer is yes automatically submit the questionnaire, if the answer is no or maybe go to the next question)

***What are the reasons participants provided for responding "No" or "Not sure" regarding intent to vaccinate your children?**

	Yes	No
Q3 The vaccine has not been adequately tested on children		
Q4 I will not give my child a vaccine that I do not know nothing about		
Q5 I don't think my child is at risk of being infected with COVID-19 so I will not give him/her the vaccine		
Q6 I am scared to put foreign object inside my children body		
Q7 Vaccination may cause infected disease		
Q8 If the government recommended it use, I will not give it to my children		
Q9 There is no way I trust big pharmaceutical company		
Q10 I believe that this virus was developed by the governments and I won't give my children any vaccine		
Q11 Vaccine cause autism		
